# A case-control study of glycemic index, glycemic load and dietary fiber intake and risk of adenocarcinomas and squamous cell carcinomas of the esophagus: the Australian Cancer Study

**DOI:** 10.1186/1471-2407-14-877

**Published:** 2014-11-24

**Authors:** Petra H Lahmann, Torukiri I Ibiebele, Penelope M Webb, Christina M Nagle, David C Whiteman, for the Australian Cancer Study

**Affiliations:** Population Health Department, QIMR Berghofer Medical Research Institute, 300 Herston Road, Herston, Brisbane, QLD 4006 Australia; School of Population Health, University of Queensland, Herston, QLD 4006 Australia

**Keywords:** Esophageal cancer, Glycemic index/load, Fiber intake

## Abstract

**Background:**

Glycemic index (GI) and glycemic load (GL) have been investigated as etiologic factors for some cancers, but epidemiological data on possible associations between dietary carbohydrate intake and esophageal cancer are scant. This study examined the association between GI, GL, and other dietary carbohydrate components and risk of adenocarcinomas and squamous cell carcinoma of the esophagus accounting for established risk factors.

**Methods:**

We analyzed data from a population-based Australian case-control study (2002-05) comprising 299 adenocarcinoma (EAC), 337 gastro-esophageal junction adenocarcinoma (EGJAC), 245 squamous cell carcinoma (ESCC), and 1507 controls sampled from a population registry. Dietary information was obtained using a 135-item food frequency questionnaire (FFQ); GI and GL were derived from an Australian GI database. Multivariable logistic regression models were used to derive odds ratios (ORs).

**Results:**

All three case groups tended to have a lower intake of fiber, and significantly higher intake of fat, total energy, and alcohol (ESCC only) compared to controls. GI was unrelated to all histological types. Higher GL was not associated with risk of EAC and EGJAC, but was inversely associated with risk of ESCC (adjusted model, p_trend_ = 0.006), specifically among men where we observed a 58% reduced risk of ESCC in the highest versus the lowest quartile. Increased intake of total carbohydrates and starch was related to similarly large risk reductions of ESCC. Fiber intake was strongly and inversely associated with risk of EAC, EGJAC and ESCC (all p_trend_ ≤0.001), indicating risk reductions of 28%-37% per 10 g/day.

**Conclusions:**

This study suggests a reduced risk of esophageal SCC with higher GL level particularly in men, but provides no evidence for the role of GI in the development of esophageal cancer. In addition, increased fiber intake appears to be associated with lower risk of all histological types of esophageal cancer.

**Electronic supplementary material:**

The online version of this article (doi:10.1186/1471-2407-14-877) contains supplementary material, which is available to authorized users.

## Background

Esophageal cancer is the eighth most common cancer worldwide, and the sixth most common cause of death from cancer [1]. The common histologic types of esophageal cancer, adenocarcinoma (EAC), gastro-esophageal junction adenocarcinoma (EGJAC) and squamous cell carcinoma (ESCC) represent different disease entities with distinct risk factor patterns [2]. While smoking, alcohol consumption and some dietary factors are the predominant risk factors for SCC, male sex, age, race, obesity and obesity-related factors are the main risk factors for EAC and EGJAC. Factors related to glucose metabolism and energy balance have been implicated in the development of a number of cancers [3, 4] and glycemic index (GI) and glycemic load (GL), both reflecting the metabolic effects of dietary carbohydrates, have been examined as possible etiologic factors [5–7]. The glycemic index ranks carbohydrate foods according to a standard food (usually glucose or white bread) based on their postprandial blood glucose response and blood insulin levels [5, 8–10]. The glycemic load combines the glycemic index value and the quantity of carbohydrate (g) to quantify the overall estimated glycemic effect of standard portion sizes of foods [8, 11]. Persistently high GI and GL intakes may lead to chronic elevations in blood glucose concentrations, while hyperglycemia, type 2 diabetes, and hyperinsulinemia have been implicated as potential risk factors for some cancers, including cancers of the digestive tract [12–14]. Further, a high-GI diet may increase cancer risk by modulating the insulin-like-growth factor (IGF) axis [15, 16]. Insulin resistance and altered levels of IGF compounds have been suggested to influence the healing of esophageal mucosal injury and esophageal cell apoptosis [12].

Results from recent meta-analyses of observational studies [6, 13] on the association between GI or GL and cancer risk, however, are mixed. Pooled risk estimates from case-control and cohort studies combined indicate a positive association between GI or GL and colorectal cancer risk, but not in cohort studies alone, and notably, no association with pancreatic or other digestive tract cancers.

Epidemiological data on dietary carbohydrate intake and esophageal cancer are scant [13, 17]. Ecological data suggest a strong correlation between carbohydrate consumption and the incidence of EAC [7]. Over the past decade two case-control studies observed slightly increased, but statistically non-significant risks for EAC [18] and ESCC [19] with higher level of GI or GL. A single prospective study [20] found that higher GI, but not GL, was significantly associated with elevated risk of esophageal cancer (EAC and ESCC cases combined) among men only. A succeeding analysis of the same cohort indicated an increased risk of esophageal adenocarcinoma with high intake of added sugars in men [21].

None of these investigations examined different histologic subtypes simultaneously to reveal any potential associations arising from their different etiologies. We therefore used data from a large population-based case-control study to examine the association between GI, GL, and other dietary carbohydrate components (total carbohydrates, starch, total sugars, fiber) and risk of EAC, EGJAC, and ESCC accounting for established risk factors and exploring potential effect modifiers.

## Patients and methods

### Study population

We used data from an Australian population-based case-control study of esophageal cancer (Australian Cancer Study, ACS) and restricted the current analysis to the group of patients who had histologically confirmed primary, invasive EAC, EGJAC or ESCC and a population-based control group. Tumors were categorized as ‘esophageal’ and ‘esophagogastric junction’ tumors according to the WHO classification [22]. Full details on the study design and recruitment have been published previously [23]. In brief, the patients (cases) were adults ages 18 to 79 years who had primary invasive carcinoma of the esophagus (ICD-10 C15) diagnosed between July 1, 2002 (July 1, 2001 in Queensland) and June 30, 2005 in the mainland states of Australia. Patients were recruited either through major treatment centers or through state-based cancer registries. Of 1,577 patients who were invited to participate in the study, 1,102 returned a completed questionnaire (70% of all those invited; 35% of all eligible patients living or deceased). Seven of these patients were deemed ineligible on pathology review and were excluded. Potential controls were selected randomly from the Australian Electoral Roll (enrolment is compulsory) within 5-year age groups and state of residence to match the distribution of the case series. Women were intentionally over sampled in the control group at all ages to accommodate their simultaneous enrolment in a parallel case-control study of ovarian cancer [24]. Of 3,042 eligible controls who were contacted, 1,580 (51%) returned completed questionnaires. For the present analyses, we excluded 152 cases and 47 control participants who did not return the food frequency questionnaire (FFQ), 35 cases and 5 controls with more than 10% of FFQ items missing, and 27 cases and 21 controls whose estimated caloric intake was extreme (<700 or >4000 kcal), leaving a final sample of 1,507 controls and 881 cases. The cases consisted of 299 (M/F 271/28) EAC cases, 337 (M/F 289/48) EGJAC cases, and 245 (M/F147/98) ESCC cases for analysis. The study was approved by the human research ethics committee of the QIMR Berghofer Institute of Medical Research and all participating institutions (Additional file 1: Table S1). All study participants provided informed written consent to take part.

### Dietary assessment

Dietary information was obtained using a 135-item semiquantitative FFQ based on the instrument developed by Willett et al. [25], but modified for use in Australia [26, 27] and validated against 12-days weighed food records [28, 29]. Assessment of our FFQ relative to the food records showed moderate correlation coefficients (r) of 0.45, 0.42, 0.53, and 0.39 for total carbohydrates, starch, total sugars, and fiber respectively for all participants [28]. Cases were asked to report their usual frequency of consumption in the year before their diagnosis or, if their diet had changed in the last 6–12 months, their usual diet. Controls were asked to report how often they consumed a specified amount of each food item in the previous year. Daily intake of energy (kcal/d), macronutrients and carbohydrate components (g/d) was estimated using Australian food composition tables as contained in NUTTAB2006 [30]. The sugar variable used was total sugars (g/d) which includes dietary mono- and disaccharides (fructose, glucose, sucrose, maltose, lactose, galactose) [30].

To calculate GL and GI, we used an Australian GI database (FoodWorks: Professional Edition, 2007) that compiled GI values based on carbohydrate-containing food items to reflect their blood glucose response. Data not available in FoodWorks were supplemented with GI values obtained from tables compiled by Atkinson and co-workers [31]. We calculated total dietary GL of a food item by multiplying the amount of carbohydrate contained in a specified serving size of the food by the quantity of that food item consumed per day and its corresponding GI value (using glucose as the reference food). We then summed the values for all carbohydrate containing foods reported on the FFQ to estimate total GL [31, 32]. The overall GI was calculated by dividing the total dietary GL by the total available carbohydrate intake.

### Covariates

Study participants provided detailed health and lifestyle information via a self-administered questionnaire [24]. Participants were asked to report their height and weight one year before diagnosis for cases and one year before study recruitment for controls. BMI (last year) was calculated as weight divided by height (kg/m^2^) and used as a predefined categorical variable according to commonly used definitions of overweight and obesity [33]. Number of pack-years of tobacco exposure was derived by dividing the number of cigarettes smoked daily by 20 and multiplying by the total number of years smoked (never smoked, <15, 15- < 30, ≥30 pack-years). Other known risk factors included in the analysis were age (y, continuous), sex (male/female), education (highschool or less, trade/diploma, university), lifetime alcohol consumption (abstainer, <0-6, 7-20, >21 standard drinks of 10 g alcohol units/week), recreational physical activity index (low, moderate, high level based on frequency and intensity of activity per week) [34], use of aspirin or other non-steroidal anti-inflammatory drugs (NSAIDs) in the last 5 years (never user, occasionally, <weekly, ≥weekly), symptoms of gastro-esophageal reflux 10 years before diagnosis (never, occasionally, <weekly, ≥weekly), presence of diabetes type 2 (no/yes, self-reported), and the following dietary factors: fruit intake (g/d), red and processed meat (g/d), and energy intake (kcal/d).

### Statistical analysis

We calculated the odds ratio (OR) and 95% confidence interval (95% CI) associated with each dietary exposure using multivariable unconditional logistic regression analysis. We combined the sexes for analysis due to small numbers of female cases, especially for esophageal adenocarcinomas. All dietary variables were adjusted for total energy intake using the nutrient residual method as described by Willett [25] and log-transformed prior to calculation of the residuals. Participants were categorized into sex-specific quartiles based on the distribution of GI, GL and other dietary carbohydrates (total carbohydrates, starch, total sugars, fiber) among the male or female controls, respectively. The first model was minimally adjusted for age and sex (data not shown). The final multivariable model was additionally adjusted for other established risk factors and other potential confounders relevant to each subtype of esophageal cancer: education, BMI last year, smoking (pack-years), lifetime mean alcohol consumption, physical activity, NSAIDs, acid reflux symptoms in last 10 years (not for ESCC), presence of diabetes (not for ESCC), and selected dietary factors. To test for linear trend across categories, the median value in each quartile was modeled as a continuous variable. Risk estimates from multivariable adjusted models were slightly attenuated, but not materially different from age and sex adjusted models; therefore results from multivariable adjusted models are presented only.

We conducted subgroup analyses to examine whether the associations between GI, GL and dietary carbohydrates were modified by sex, BMI (<25 and ≥25), smoking status (ever/never), or current alcohol consumption (</>sex-specific median g/d), red meat and saturated fat intake (</>sex-specific median g/d), diabetes type 2 (yes/no, EAC and EGJAC only), acid reflux symptoms (ever/never, EAC and EGJAC only). The statistical significance of any observed stratum differences was assessed by including a cross-product term in regression models. We present sex-stratified analysis for ESCC only in supplementary material. Further, we conducted sensitivity analysis, using a) a combined smoking variable derived from current smoking status and pack-years, b) explored various energy adjustments, c) omitted BMI from multivariable analysis for EAC. All analyses were conducted using the SAS statistical software, version 9.1 (SAS Institute, Cary, NC), and statistical tests were 2-sided with *P*-values <0.05 considered statistically significant.

## Results

Study participants characteristics by subtype of esophageal cancer are provided in Table 1. Overall, cases were predominantly male, with the highest proportion of women (40%) among ESCC cases. Compared to controls, all 3 groups of cases tended to be older on average, were less likely to have a university degree, more likely to be heavy smokers (≥30 pack years) and heavy drinkers (≥21 drinks/week, lifetime) and to have a low physical activity level (ESCC only). As expected, the proportion of obese individuals among EAC (37%) and EGJAC (33%) cases, but not ESCC (14%) cases, was substantially higher than among controls (21%). Likewise, diabetes was more common among EAC and EGJAC cases (11-12%) compared to their control counterparts (7%). Aspirin/NSAID use did not differ significantly by case status. With regard to dietary factors, all three groups of cases tended to have a lower intake of fiber and protein (ESCC only), and higher intake of total energy, total fat (g/day or % energy), saturated fat, and for ESCC cases only, higher current alcohol consumption (g/day) as compared to controls. Among all study participants, vegetables (41%), fruit (28%), and grains (23%) were the main food sources of dietary fiber (% of total intake); grains (56%), vegetables (28%), and sweet snacks (10%) of starch; and fruit (36%), sweet snacks (23%), dairy products (20%), and soft drinks (9%) of total sugars.

**Table 1:** **Non-dietary and dietary characteristics of study participants, N = 2,388**

Characteristics	Controls	EAC	P value^a^	EGJAC	P value^a^	ESCC	P value^a^
	***N***= 1507	***N***= 299		***N***= 337		***N***= 245	
Non-dietary factors							
Age (y, mean, SD)	61 (12)	64 (10)	<0.0001	63 (10)	<0.0001	65 (9)	<0.0001
Sex (%)							
Female	34	9	<0.0001	14	<0.0001	40	0.06
Male	66	91		86		60	
Educational level (%)							
Highschool or less	40.9	46.5	0.0002	37.7	0.01	57.1	<0.0001
Trade/diploma	43.6	47.2		51.6		34.3	
University degree	15.5	6.3		10.7		8.6	
Body mass index (kg/m^2^, %)						s	
< 25	36.2	20.5	<0.0001	27.4	< 0.0001	55.5	<0.0001
25 - <30	43.3	42.7		39.2		30.4	
≥ 30	20.5	36.8		33.4		14.1	
Physical activity index (%)							
Low	19.3	23.8	0.33	21.7	0.76	29.5	0.0003
Moderate	41.0	36.6		37.7		30.7	
High	39.7	39.6		40.6		39.8	
Cumulative smoking history (pack-years, %)							
Never smoked	44.7	25.1	<0.0001	24.3	< 0.0001	23.1	<0.0001
< 15	25.2	20.1		19.9		19.7	
15-29.9	13.3	19.1		22.3		20.2	
≥ 30	16.8	35.7		33.5		37.0	
Lifetime alcohol consumption (10 g alcohol units/wk) (%)							
Abstainer	10.7	6.4	<0.0001	9.2	0.0004	12.7	<0.0001
> 0-6 drinks/wk	38.1	27.8		29.7		26.6	
7-20 drinks/wk	31.9	36.6		33.5		20.9	
≥ 21 drinks/wk	19.3	29.2		27.6		39.8	
Reflux symptoms 10 years ago (%)							
Never	43.1	21.5	<0.0001	29.7	<0.0001	46.0	<0.0001
Occasionally	30.5	13.5		16.1		13.0	
< Weekly	14.5	22.6		22.6		13.0	
≥ Weekly	11.9	42.4		31.6		28.0	
NSAID use (%)							
Never user	43.7	46.3	0.24	47.6	0.62	51.4	0.07
Occasionally	31.4	26.0		28.7		27.4	
< Weekly	9.0	8.8		9.0		7.5	
≥ Weekly	15.9	18.9		14.7		13.7	
Presence of Diabetes (%)							
No	93.0	87.6	0.002	88.2	0.02	95.8	0.09
Yes	7.0	12.4		11.2		4.2	
Dietary factors (Mean, SD)^b^							
Glycemic Index^c^	51 (5)	52 (5)	0.38	52 (5)	0.16	52 (6)	0.17
Glycemic load^c^	120 (23)	119 (22)	0.41	118 (22)	0.18	115 (25)	0.002
Total carbohydrates (g)^c^	234 (34)	231 (34)	0.14	229 (31)	0.01	221 (37)	<0.0001
Total sugars (g)^c^	128 (35)	127 (33)	0.49	124 (33)	0.05	117 (38)	<0.0001
Starch (g)^c^	101 (24)	98 (25)	0.04	99 (24)	0.28	94 (27)	0.001
Dietary Fiber (g)^c^	31 (9)	28 (8)	<0.0001	27 (8)	< 0.0001	28 (9)	<0.0001
Protein (g)	93 (15)	91 (14)	0.25	92 (15)	0.29	89 (17)	0.003
Fat (g)	71 (12)	73 (12)	0.04	74 (11)	0.001	73 (13)	0.03
Saturated fat (g)	27 (7)	29 (7)	<0.0001	30 (7)	<0.0001	29 (7)	0.0005
MUFA (g)	25 (6)	25 (5)	0.65	26 (5)	0.02	25 (6)	0.67
PUFA (g)	11 (3)	10 (3)	0.002	11 (3)	0.48	11 (3)	0.80
Alcohol consumption (g)^d^	13 (16)	14 (16)	0.20	13 (17)	0.78	21 (24)	<0.0001
Total Energy (kcal)	2215 (644)	2395 (729)	<0.0001	2396 (700)	<0.0001	2270 (795)	<0.0001
% energy from CHO	45 (7)	45 (7)	0.23	45 (6)	0.03	43 (7)	<0.0001
% energy from protein	18 (3)	18 (7)	0.06	18 (3)	0.08	17 (3)	0.003
% energy from fat	30 (5)	31 (5)	0.02	31 (5)	<0.0001	31 (5)	0.03

^a^p-level chi-square test for categorical variables or chi-square test for trend, and *t*-test for continuous variables.

^b^dietary variables adjusted for energy intake (nutrient residual method), except for GI.

^c^Median values for dietary exposure variables by sex (male/female controls): GI 52/50, GL 121/116, total carbohydrates 234/236 g/d, total sugars 123/132 g/d, starch 100/100 g/d, dietary fiber 29/32 g/d.

^d^current alcohol consumption (FFQ).

Tables 2 and 3 present adjusted ORs for each subtype of esophageal cancer according to intakes of GI, GL, carbohydrate components and fiber for men and women combined. GI and GL were not associated with risk of EAC or EGJAC (Table 2), whereas higher GL was associated significantly and inversely with risk of ESCC (Table 3) in the fully adjusted model (p_trend_ = 0.006). We observed a 48% reduced risk of ESCC in the highest quartile compared with the lowest (reference) quartile. GI was unrelated to risk of ESCC. In sensitivity analyses (not shown), we additionally adjusted for fiber intake in the multivariable models. Risk estimates were not materially different for any case group. Further, to test for confounding or mediating effects of diabetes or BMI, we conducted sensitivity analyses especially for adenocarcinomas (not shown). We excluded individuals with diabetes in each dietary exposure multivariable model, and separately, for total sugars intake only, we omitted BMI from the multivariable model. ORs were not significantly changed in any of these analyses.

**Table 2:** **Odds ratios and 95% confidence intervals for esophageal adenocarcinomas and gastro-esophageal junction adenocarcinomas according to glycemic index, glycemic load, and dietary carbohydrate intakes, Australia 2002-2005**

	Controls	EAC		EGJAC	
		Multivariable model^a^		Multivariable model^a^	
	N = 1490	N = 288	OR, (95% CI)	N = 318	OR, (95% CI)
Glycemic Index (median, range)^b^					
Q1: 46 (27-49)	374	67	1.00	69	1.00
Q2: 50 (49-51)	374	64	0.89 (0.59-1.36)	74	0.98 (0.67-1.45)
Q3: 53 (51-54)	368	80	0.91 (0.61-1.37)	94	1.07 (0.73-1.56)
Q4: 57 (54-71)	374	77	0.82 (0.54-1.26)	81	0.78 (0.52-1.18)
P-trend^c^			0.42		0.33
Per 10 unit/day increment			0.81 (0.58-1.12)		*0.83 (0.61-1.13)*
Glycemic Load (median, range)^b^					
Q1: 95 (21-105)	373	74	1.00	82	1.00
Q2: 113 (105-120)	375	77	1.03 (0.69-1.54)	100	1.15 (0.80-1.65)
Q3: 126 (120-135)	371	77	0.88 (0.59-1.31)	68	0.75 (0.51-1.11)
Q4: 146 (135-259)	371	60	0.73 (0.48-1.13)	68	0.72 (0.49-1.08)
P-trend^c^			0.12		0.03
Per 50 unit/day increment			0.83 (0.60-1.16)		*0.78 (0.57-1.07)*
Carbohydrate (g/day) (median, range)^b^					
Q1: 196 (94-212)	372	79	1.00	87	1.00
Q2: 224 (212-235)	375	82	1.14 (0.77-1.68)	97	1.21 (0.84-1.73)
Q3: 245 (235-256)	374	69	0.90 (0.59-1.38)	76	0.95 (0.64-1.40)
Q4: 273 (256-438)	369	58	0.79 (0.49-1.25)	58	0.75 (0.48-1.16)
P-trend^c^			0.21		0.13
Per 50 g/day increment			0.94 (0.74-1.21)		*0.87 (0.69-1.09)*
Starch (g/day) (median, range)^b^					
Q1: 74 (31-85)	372	85	1.00	90	1.00
Q2: 93 (85-100)	374	73	1.00 (0.68-1.49)	79	0.90 (0.62-1.31)
Q3: 107 (100-116)	372	66	0.89 (0.59-1.33)	84	0.99 (0.69-1.43)
Q4: 128 (116-249)	372	64	0.80 (0.53-1.21)	65	0.71 (0.48-1.06)
P-trend^c^			0.25		0.17
Per 50 g/day increment			0.85 (0.63-1.16)		*0.86 (0.64-1.14)*
Total sugars (g/day) (median, range)^b^					
Q1: 90 (25-106)	374	74	1.00	97	1.00
Q2: 116 (106-126)	371	71	1.10 (0.73-1.67)	84	1.01 (0.69-1.46)
Q3: 136 (126-148)	374	68	1.18 (0.76-1.84)	67	0.90 (0.60-1.34)
Q4: 168 (148-395)	371	75	1.22 (0.77-1.92)	70	0.88 (0.58-1.35)
P-trend^c^			0.38		0.47
Per 50 g/day increment			1.08 (0.85-1.36)		*1.01 (0.81-1.27)*
Fiber (g/day) (median, range)^b^					
Q1: 21 (7-25)	371	106	1.00	122	1.00
Q2: 28 (25-30)	374	77	0.78 (0.54-1.14)	99	0.91 (0.65-1.27)
Q3: 33 (30-36)	372	58	0.63 (0.41-0.95)	68	0.63 (0.43-0.91)
Q4: 41 (36-74)	373	47	0.49 (0.31-0.77)	36	0.34 (0.21-0.54)
P-trend^c^			0.001		<.0001
Per 10 g/day increment			0.72 (0.59-0.87)		*0.63 (0.53-0.76)*

^a^Multivariable Model: adjusted for age, sex, education, BMI, smoking (pack years), physical activity, lifetime mean alcohol intake, acid reflux symptoms in last 10 years, non-steroidal anti-inflammatory drug (NSAID) use, presence of diabetes, total fruit intake (except for fiber intake), red meat, processed meat, and total energy.

^b^Sex-specific quartile cut-off points are: glycemic index 47, 50, 53 for women and 49, 52, 55 for men; glycemic load 102, 117, 132 for women and 106, 121, 136 for men; carbohydrate 215, 237, 259 for women and 211, 234, 255 for men; starch 85, 100, 114 for women and 85, 100, 117 for men; sugar 115, 132, 151 for women and 102, 123, 145 for men; fiber 27, 32, 39 for women and 24, 29, 35 for men.

^c^ Likelihood ratio test for trend across dietary variables quartiles by using an ordinal variable coded as the median value of the quartile.

**Table 3:** **Odds ratios and 95% confidence intervals for esophageal squamous cell carcinoma according to glycemic index, glycemic load, and dietary carbohydrate intakes, Australia 2002-2005**

	Controls	ESCC	
		Multivariable model^a^	
	N = 1490	N = 227	OR, (95% CI)
Glycemic Index (median, range)^b^			
Q1: 46 (27-49)	374	60	1.00
Q2: 50 (49-51)	374	39	0.66 (0.41-1.05)
Q3: 53 (51-54)	368	58	1.01 (0.66-1.56)
Q4: 57 (54-71)	374	70	0.73 (0.46-1.14)
P-trend^c^			0.44
Per 10 unit/day increment			0.89 (0.66-1.21)
Glycemic Load (median, range)^b^			
Q1: 95 (21-105)	373	85	1.00
Q2: 113 (105-120)	375	52	0.61 (0.40-0.92)
Q3: 126 (120-135)	371	48	0.67 (0.44-1.02)
Q4: 146 (135-259)	371	42	0.52 (0.33-0.82)
P-trend^c^			0.006
Per 50 unit/day increment			0.70 (0.50-0.97)
Carbohydrate (g/day) (median, range)^b^			
Q1: 196 (94-212)	372	98	1.00
Q2: 224 (212-235)	375	58	0.81 (0.54-1.21)
Q3: 245 (235-256)	374	35	0.55 (0.35-0.87)
Q4: 273 (256-438)	369	36	0.46 (0.28-0.75)
P-trend^c^			0.0005
Per 50 g/day increment			0.72 (0.56-0.92)
Starch (g/day) (median, range)^b^			
Q1: 74 (31-85)	372	91	1.00
Q2: 93 (85-100)	374	49	0.55 (0.36-0.84)
Q3: 107 (100-116)	372	44	0.52 (0.34-0.80)
Q4: 128 (116-249)	372	43	0.46 (0.29-0.71)
P-trend^c^			0.0003
Per 50 g/day increment			0.60 (0.43-0.82)
Total sugars (g/day) (median, range)^b^			
Q1: 90 (25-106)	374	97	1.00
Q2: 116 (106-126)	371	35	0.54 (0.34-0.85)
Q3: 136 (126-148)	374	47	0.79 (0.51-1.23)
Q4: 168 (148-395)	371	48	0.83 (0.53-1.32)
P-trend^c^			0.59
Per 50 g/day increment			0.86 (0.68-1.09)
Fiber (g/day) (median, range)^b^			
Q1: 21 (7-25)	371	93	1.0
Q2: 28 (25-30)	374	53	0.64 (0.43-0.97)
Q3: 33 (30-36)	372	45	0.45 (0.29-0.71)
Q4: 41 (36-74)	373	36	0.38 (0.23-0.63)
P-trend^c^			<.0001
Per 10 g/day increment			0.64 (0.52-0.79)

^a^Multivariable Model: adjusted for age, sex, education, BMI, smoking (pack years), physical activity, lifetime mean alcohol intake, non-steroidal anti-inflammatory drug (NSAID) use, total fruit intake (except for fiber intake), red meat, processed meat, and total energy.

^b^Sex-specific quartile cut-off points are: glycemic index 47, 50, 53 for women and 49, 52, 55 for men; glycemic load 102, 117, 132 for women and 106, 121, 136 for men; carbohydrate 215, 237, 259 for women and 211, 234, 255 for men; starch 85, 100, 114 for women and 85, 100, 117 for men; sugar 115, 132, 151 for women and 102, 123, 145 for men; fiber 27, 32, 39 for women and 24, 29, 35 for men.

^c^Likelihood ratio test for trend across dietary variables quartiles by using an ordinal variable coded as the median value of the quartile.

Total carbohydrate intake or selected carbohydrate components were not related to risk of EAC and EGJAC (Table 2). In contrast, mean intake of total carbohydrate and starch was associated with similarly large risk reductions (54%) of ESCC (Table 3), when comparing highest with lowest quartile (both p _trend_ <0.0006). Mean fiber intake was strongly and inversely associated with risk of EAC, EGJAC, and ESCC (all p _trend_ <0.001). Specifically, the cancer risk for each subtype was reduced between 28%-37% per 10 g/day increment of fiber intake (OR, 95%CI: EAC 0.72, 0.59-0.87; EGJAC 0.63, 0.53-0.76; ESCC 0.64, 0.52-0.79).

**Figure 1:**
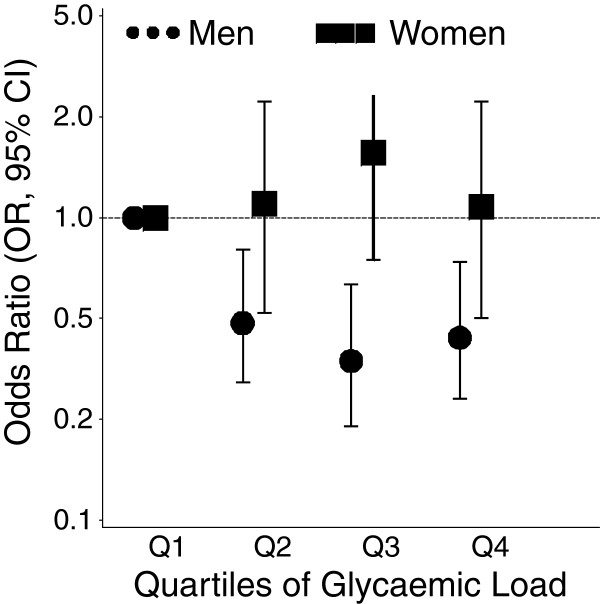
**Multivariable-adjusted odds ratios (ORs, CI 95%) of esophageal squamous cell carcinoma (plotted on logarithmic scale) are illustrated for men and women according to quartiles of Glycemic Load (sex-specific quartile cut-off points: men 106, 121, 136; women 102, 117, 132).**

To assess potential effect modification of the GI/GL-esophageal cancer association by selected covariates, interaction terms for each glycemic/carbohydrate factor with sex, BMI, smoking status or alcohol consumption were tested. None of the tested interactions were statistically significant with few exceptions. We observed that the association between GL and ESCC was modified by sex (p _interaction_ = 0.02) presented in Figure 1. The inverse risk pattern was confined to men only (OR, 95%CI; quartile 2: 0.48, 0.28-0.80; quartile 3: 0.34, 0.19-0.63; quartile 4: 0.42, 0.24-0.74), while no clear association became apparent among women (OR, 95%CI; quartile 2: 1.13, 0.53-2.38; quartile 3: 1.61, 0.77-3.39; quartile 4: 1.07, 0.50-2.32) as shown in sex-specific Additional file 2: Table S2 and Additional file 3: Table S3. Similar to GL, the carbohydrates and starch associations differed by sex (total carbohydrates: p _interaction_ = 0.02; starch: p _interaction_ = 0.03). The decreased risk of ESCC was accentuated in men only (Additional file 2: Table S2 and Additional file 3: Table S3). Further, we examined the potential effect modification of the GI/GL/carbohydrate component-esophageal cancer association by red meat intake and saturated fat intake (data not shown). There was no evidence that these dietary factors modified the association between GI or GL and any subtype. For ESCC only, in stratified analysis, the inverse total carbohydrates association remained only among those with high red meat intake (median split: >91 g/d, p _trend_ = 0.0005), while the inverse fiber association was confined to those with low fat intake (median split: <26.5 g/d, p _trend_ <0.0001).

## Discussion

In this large case-control study of Australian men and women, GI was unrelated to risk of all histologic types of esophageal cancer. GL was not associated with risk of EAC and EGJAC, but was inversely associated with risk of ESCC (30% risk reduction per 10 unit/d increment). This dose-dependent association was independent of other established risk factors, including smoking status, alcohol consumption, BMI and selected dietary factors. Sex-stratified analysis indicated that this association was confined to men only. Similar to GL, higher intakes of total carbohydrates and starch were significantly related to lowered risk of ESCC. Total dietary fiber intake was inversely and strongly associated with all three tumor types independent of sex (all p _trend_ ≤0.001).

While published data on colorectal cancer suggest a small to moderate increased risk with higher GI or GL [6, 13, 20], findings derived from the few published reports on esophageal cancer are not clear and reported associations are of low magnitude. Results based on the prospective National Institutes of Health (NIH)-AARP Diet and Health Study [20] indicate that among men, higher GI, but not GL, was associated with increased risk of esophageal cancers (adenocarcinoma and squamous cell carcinoma combined, 425 cases). Interestingly, in stratified analyses, this association remained significant only among smokers (former/current), men with a high BMI, or high saturated fat intake. The FINBAR case-control study [18] including 224 EAC cases (84% men), showed a 42% increased risk per 10 unit higher GI intake for this tumor type, and appeared to be stronger (but not significantly) in centrally overweight individuals. An earlier hospital-based case-control study [19] including 304 ESCC cases (90% men), suggested borderline significant direct associations between GI (OR (95%CI) 1.1, 0.9-1.5, per 10-unit/d increment) or GL (1.2, 1.0-1.5, per 100-unit/d increment) and ESCC risk.

Our observations made in the present study differ from previous evidence in that glycemic indicators seem to have a higher impact on risk of ESCC than on either type of adenocarcinoma of the esophagus, and rather GL, not GI, had a relevant effect on cancer risk. The latter finding supports the suggestion by Hu et al. [35] that GL is a more physiologically relevant measure than GI in terms of associated risk with chronic disease. Further, because of the narrow distribution of GI values (27-71, median 52) in this study population which centered around the middle of the theoretical range for GI (0-100), we may have not been able to detect significant effects of different levels of GI. This issue has also been raised by other investigators [20, 36]. Moreover, in contrast to the NIH-AARP Diet and Health Study [20] we did not observe any effect modification by smoking status (ever/never), BMI (<25>), or saturated fat intake (median split) on the GI/GL-esophageal cancer association (data not shown).

We have no straightforward explanation for the observed moderate inverse association between GL and ESCC risk which was observed only among men after stratification by sex. It has been suggested that the direction and magnitude of glycemic indicators-cancer associations may be explained by the way in which high GI or high GL track with other dietary and lifestyle factors related to cancer development [20]. For instance, in the NIH-AARP cohort, high GL diets were inversely related to total cancer only among adults with low BMI [20]. This is compatible with our finding of the inverse GL-esophageal cancer association among ESCC cases only, who on average have lower BMI than their counterparts diagnosed with EAC or EGJAC as documented in this and our previous studies [23, 37]. Considering each tumor type separately, however, the GI/GL-esophageal cancer association was consistent across all BMI levels; hence BMI did not modify the relation between GI or GL and any of the histologic types.

Higher fiber intake was associated with reduced risks of all three tumor types in our investigation (28-37% risk reduction per 10-unit/d increment). This is in accordance with other population-based case-control studies demonstrating an inverse association between dietary fiber and risk of EAC [18, 38–42], EGJAC [40–42], and ESCC [40]. Based on our findings, no obvious heterogeneity of the association between fiber and adenocarcinoma and squamous cell carcinoma of the esophagus became apparent, which is similar to one previous report [40], but contrasts with another [41]. In the latter study total dietary fiber intake was significantly related to gastric cardia adenocarcinoma only. A recent meta-analysis on dietary fiber and esophageal cancer risk, including a total of 10 population-based or hospital-based case-control studies, also indicates a more consistent inverse association for EAC than for ESCC [43]. When exploring potential effect modification by selected dietary factors, we observed that the inverse fiber-ESCC association was confined to those individuals with lower fat intake.

Fiber has a potential role in cancer prevention by beneficially influencing blood glucose control, lipid profiles, and body weight [44–46]. Although the protective mechanism of fiber is not well understood, it may act by mechanical removal of carcinogens from food items that pass through the digestive tract and/or removal of damaged cells from the epithelial surface, by lowering plasma levels of biomarkers of systemic inflammation, and by reducing risk of hiatus hernia and gastro-esophageal reflux symptoms, or by mediating the glycemic response as summarized by others [12, 18, 40, 43]. However, similar to overall carbohydrate intake, high fiber intake may be a proxy for a diet rich in other bioactive constituents (e.g. micronutrients) that are protective against cancer, including esophageal malignancies.

Some limitations warrant consideration when interpreting results of our study. A potential shortcoming was the low participation rate among controls, which increases the likelihood that our control sample was not representative of the population from which the cases arose. To assess the magnitude of possible bias, we compared smoking and obesity prevalence in the control group with that reported in the 2004 Australian National Health Survey (NHS) [47]. The prevalence of ever-smoking and the distribution of BMI in our study were similar to those in the NHS, and using the NHS distributions to impute BMI values for nonparticipating controls had minimal effect on risk estimates [48]. Dietary measurement errors may have occurred in our dietary assessment, including nondifferential misclassification of exposure, and dietary recall bias related to cancer status, BMI and possibly other relevant exposures all of which would attenuate effect estimates [49, 50]. It is likely that systematic error may be present due to misreporting of energy- and macronutrient intake by BMI status [51] specifically selective underreporting by overweight women [52]. Other limitations include the possibility of residual confounding from smoking, alcohol consumption, and unmeasured variables. We have not validated the assessment of GI or GL against an objective standard or using a different dietary method. However, average GI and GL intake values of female and male participants in our study are compatible with dietary data from other Australian studies assessed by FFQ [53, 54] or diet history interview [55].

Major strengths of our population-based study include its large sample size, the examination of three different but related esophageal cancer endpoints, a high case-response rate, and the comprehensive control of other risk factors.

## Conclusions

In conclusion, this case-control study in Australian adults suggests a reduced risk of esophageal SCC with higher GL level, most notably among men, but provides no evidence for the role of a high GI diet in the development of adenocarcinomas or squamous cell carcinomas of the esophagus. Increased total fiber intake appeared to be comparably protective for all histological types. This finding is in accordance with previous evidence from case-control studies on esophageal cancers. Given the limited number of epidemiological studies on glycemic indicators and risk of adenocarcinomas and squamous cell carcinoma of the esophagus, it remains to be shown whether GI and/or GL are meaningful predictors of these malignancies.

## Electronic supplementary material

Additional file 1: Table S1: The Australian Cancer Study, names of the ethics committees from all institutions that approved this study. (DOC 58 KB)

Additional file 2: Table S2: Odds ratios and 95% confidence intervals for esophageal squamous cell carcinoma according to glycemic index, glycemic load, and dietary carbohydrate intakes in men, Australia 2002-2005. (DOCX 22 KB)

Additional file 3: Table S3: Odds ratios and 95% confidence intervals for esophageal squamous cell carcinoma according to glycemic index, glycemic load, and dietary carbohydrate intakes in women, Australia 2002-2005. (DOCX 21 KB)
