# Supplementary material for: A case-control study of glycemic index, glycemic load and dietary fiber intake and risk of adenocarcinomas and squamous cell carcinomas of the esophagus: the Australian Cancer Study
Source: BMC Cancer. 2014 Nov 24;14:877. doi: 10.1186/1471-2407-14-877 (PMC4255966; doi:10.1186/1471-2407-14-877)
Supplement: Supplementary file 1 — Additional file 1: Table S1: The Australian Cancer Study, names of the ethics committees from all institutions that approved this study. (DOC 58 KB) [file 12885_2014_5054_MOESM1_ESM.doc]

Additional Table 1: The Australian Cancer Study, names of the ethics committees from all institutions that approved this study

| Name of Site | Human Research Ethics Committee (HREC) |
| --- | --- |
| QUEENSLAND (QLD) |  |
| The Queensland Institute of Medical Research  (now QIMR Berghofer Medical Research Institute) | Bancroft Centre Research Ethics Committee |
| University of Queensland | Medical Research Ethics Committee |
| Mater Misericordiae Hospitals | Human Research Ethics Committee |
| Princess Alexandra Hospital | PAH Human Research Ethics Committee |
| Royal Brisbane Hospital | RBH Research Ethics Committee |
| Ipswich Hospital | West Moreton Health Service District Human Research Ethics Committee |
| Gold Coast Hospital | Research Ethics Committee |
| Logan Hospital | Logan Hospital Executive |
| Rockhampton Hospital | Rockhampton Health Service District Human Research Ethics Committee |
| Nambour Hospital | (Medical Director approval) |
| Townsville General Hospital | Townsville Health Service District Institutional Ethics Committee |
| Queen Elizabeth II Jubilee Hospital | (Medical Director Approval) |
| WESTERN AUSTRALIA (WA) |  |
| Royal Perth Hospital | Royal Perth Hospital Ethics Committee  (reciprocal arrangements with other Perth hospitals) |
| Sir Charles Gairdner Hospital | Human Research Ethics Committee |
| Fremantle Hospital | Human Research Ethics Committee |
| St John of God Hospital | St John of God Health Care Ethics Committee |
| The Mount Hospital | Mt Hospital HREC |
| SOUTH AUSTRALIA (SA) |  |
| Queen Elizabeth Hospital | North Western Adelaide Health Service Ethics of Human Research Committee |
| Flinders Medical Centre | Flinders Clinical Research Ethics Committee |
| Royal Adelaide Hospital | Royal Adelaide Research Ethics Committee |
| NEW SOUTH WALES (NSW) |  |
| NSW | NSW Cancer Council Research Ethics Committee |
| Royal North Shore Hospital | Northern Sydney Health Human Research Ethics Committee |
| The Nepean Hospital | Wentworth Area Health Services Ethics Committee |
| St George Hospital | South East Health Human Research ethics Committee |
| Concord Hospital | CSAHS HREC |
| Royal Prince Alfred Hospital | RPA Ethics Research Committee |
| Hunter Hospital | Hunter New England Human Research Ethics Unit |
| Liverpool Hospital | SWSAHS |
| St Vincents Hospital | St Vincents Human Research Ethics Committee |
| Westmead Hospital | WSAHS |
| AUSTRALIAN CAPITAL TERRITORY (ACT) |  |
| Canberra Hospital | ACT Health Ethics Committee |
| VICTORIA (VIC) |  |
| VIC | Cancer Council Victoria Human Research Ethics Committee |
| Peter MacCallum Cancer Institute | Peter MacCallum Cancer Institute Research Institute Ethics Committee |
| Royal Melbourne Hospital | Royal Melbourne Ethics Committee |
| Monash Medical Centre | Southern Health Research Ethics Committee |
| Alfred Hospital | Alfred Research & Ethics Committee |
| Geelong Hospital  (St John of God) | Barwon Health Research & Ethics Committee |
| Box Hill Hospital | Eastern Health HREC |
| Cabrini Hospital | Cabrini Human Research Ethics Committee |
| St Vincents Hospital | St Vincents HREC |
| The Austin and Repatriation Medical Centre | Human Research Ethics Committee  Austin Campus |
