# Supplementary material for: A case-control study of glycemic index, glycemic load and dietary fiber intake and risk of adenocarcinomas and squamous cell carcinomas of the esophagus: the Australian Cancer Study
Source: BMC Cancer. 2014 Nov 24;14:877. doi: 10.1186/1471-2407-14-877 (PMC4255966; doi:10.1186/1471-2407-14-877)
Supplement: Supplementary file 2 — Additional file 2: Table S2: Odds ratios and 95% confidence intervals for esophageal squamous cell carcinoma according to glycemic index, glycemic load, and dietary carbohydrate intakes in men, Australia 2002-2005. (DOCX 22 KB) [file 12885_2014_5054_MOESM2_ESM.docx]

Additional table 2

Odds ratios and 95% confidence intervals for esophageal squamous cell
carcinoma according to glycemic index, glycemic load, and dietary carbohydrate
intakes in men, Australia 2002-2005

|  | Controls  (n=983) | Cases  (n=138) | OR, (95% CI)  Multivariable model^a^ |
| --- | --- | --- | --- |
| Glycaemic Index (median, range) |  |  |  |
| Q1: 47 (29-49) | 245 | 37 | 1.0 |
| Q2: 51 (49-52) | 247 | 23 | 0.52 (0.29-0.95) |
| Q3: 53 (52-55) | 244 | 31 | 0.71 (0.41-1.27) |
| Q4: 57 (55-70) | 247 | 47 | 0.63 (0.36-1.09) |
| P-trend^b^ |  |  | 0.22 |
| *Per 10 unit/day increment* |  |  | *0.71 (0.47-1.07)* |
| Glycaemic Load (median, range) |  |  |  |
| Q1: 96 (52-106) | 245 | 62 | 1.0 |
| Q2: 114 (106-121) | 248 | 31 | 0.48 (0.29-0.81) |
| Q3: 127 (121-136) | 244 | 18 | 0.34 (0.19-0.64) |
| Q4: 146 (136-259) | 246 | 27 | 0.42 (0.24-0.74) |
| P-trend^b^ |  |  | 0.0006 |
| *Per 50 unit/day increment* |  |  | *0.56 (0.36-0.85)* |
| Carbohydrate (g/day) (median, range) |  |  |  |
| Q1: 193 (129-210) | 245 | 68 | 1.0 |
| Q2: 222 (211-234) | 247 | 32 | 0.64 (0.39-1.08) |
| Q3: 245 (234-255) | 247 | 22 | 0.54 (0.30-0.95) |
| Q4: 271 (255-438) | 244 | 16 | 0.32 (0.16-0.63) |
| P-trend^b^ |  |  | 0.0006 |
| *Per 50 g/day increment* |  |  | *0.63 (0.46-0.86)* |
| Starch (g/day) (median, range) |  |  |  |
| Q1: 74 (31-85) | 245 | 65 | 1.0 |
| Q2: 93 (85-100) | 247 | 27 | 0.46 (0.27-0.79) |
| Q3: 108 (100-117) | 246 | 26 | 0.49 (0.28-0.84) |
| Q4: 128 (117-201) | 245 | 20 | 0.35 (0.19-0.64) |
| P-trend^b^ |  |  | 0.0004 |
| *Per 50 g/day increment* |  |  | *0.47 (0.30-0.73)* |
| Sugar (g/day) (median, range) |  |  |  |
| Q1: 87 (25-106) | 246 | 65 | 1.0 |
| Q2: 113 (102-123) | 247 | 26 | 0.56 (0.32-0.95) |
| Q3: 133 (123-145) | 244 | 22 | 0.63 (0.35-1.12) |
| Q4: 165 (145-395) | 246 | 25 | 0.67 (0.37-1.21) |
| P-trend^b^ |  |  | 0.14 |
| *Per 50 g/day increment* |  |  | *0.81 (0.60-1.10)* |
| Fibre (g/day) (median, range) |  |  |  |
| Q1: 20 (7-24) | 245 | 69 | 1.0 |
| Q2: 27 (24-29) | 246 | 29 | 0.58 (0.35-0.98) |
| Q3: 32 (29-35) | 246 | 19 | 0.35 (0.20-0.64) |
| Q4: 39 (35-69) | 246 | 21 | 0.46 (0.25-0.85) |
| P-trend^b^ |  |  | 0.001 |
| *Per 10 g/day increment* |  |  | *0.59 (0.45-0.77)* |

^a^ Multivariable Model: adjusted for age, education, BMI, smoking (pack years), physical

activity, lifetime mean alcohol intake, non-steroidal anti-inflammatory drug (NSAID) use, total

fruit intake (except for fiber intake), red meat, processed meat, and total energy

^b^ Likelihood ratio test for trend across dietary variables quartiles by using an ordinal variable

coded as the median value of the quartile
