# Supplementary material for: A case-control study of glycemic index, glycemic load and dietary fiber intake and risk of adenocarcinomas and squamous cell carcinomas of the esophagus: the Australian Cancer Study
Source: BMC Cancer. 2014 Nov 24;14:877. doi: 10.1186/1471-2407-14-877 (PMC4255966; doi:10.1186/1471-2407-14-877)
Supplement: Supplementary file 3 — Additional file 3: Table S3: Odds ratios and 95% confidence intervals for esophageal squamous cell carcinoma according to glycemic index, glycemic load, and dietary carbohydrate intakes in women, Australia 2002-2005. (DOCX 21 KB) [file 12885_2014_5054_MOESM3_ESM.docx]

Additional table 3

Odds ratios and 95% confidence intervals for esophageal squamous cell
carcinoma according to glycemic index, glycemic load, and dietary carbohydrate
intakes in women, Australia 2002-2005

|  | Controls  (n=507) | Cases  (n=89) | OR, (95% CI)  Multivariable model^a^ |
| --- | --- | --- | --- |
| Glycaemic Index (median, range) |  |  |  |
| Q1: 45 (27-47) | 128 | 28 | 1.0 |
| Q2: 49 (47-50) | 127 | 7 | 0.27 (0.10-0.71) |
| Q3: 52 (50-53) | 124 | 25 | 1.12 (0.56-2.24) |
| Q4: 55 (53-71) | 128 | 29 | 0.89 (0.43-1.82) |
| P-trend ^b^ |  |  | 0.64 |
| *Per 10 unit/day increment* |  |  | *1.30 (0.79-2.15)* |
| Glycaemic Load (median, range) |  |  |  |
| Q1: 95 (21-102) | 126 | 23 | 1.0 |
| Q2: 109 (102-117) | 129 | 20 | 1.13 (0.53-2.38) |
| Q3: 123 (117-132) | 126 | 25 | 1.61 (0.77-3.39) |
| Q4: 145 (132-235) | 126 | 21 | 1.07 (0.50-2.32) |
| P-trend ^b^ |  |  | 0.63 |
| *Per 50 unit/day increment* |  |  | *1.02 (0.60-1.75)* |
| Carbohydrate (g/day) (median, range) |  |  |  |
| Q1: 200 (94-215) | 126 | 29 | 1.0 |
| Q2: 227 (215-237) | 128 | 26 | 1.30 (0.65-2.61) |
| Q3: 245 (237-259) | 128 | 15 | 0.70 (0.32-1.56) |
| Q4: 275 (259-341) | 125 | 19 | 0.75 (0.35-1.62) |
| P-trend ^b^ |  |  | 0.26 |
| *Per 50 g/day increment* |  |  | *0.91 (0.60-1.38)* |
| Starch (g/day) (median, range) |  |  |  |
| Q1: 74 (32-85) | 126 | 26 | 1.0 |
| Q2: 93 (85-100) | 128 | 21 | 0.81 (0.39-1.70) |
| Q3: 106 (100-113) | 127 | 19 | 0.70 (0.33-1.47) |
| Q4: 126 (114-249) | 126 | 23 | 0.63 (0.30-1.23) |
| P-trend ^b^ |  |  | 0.20 |
| *Per 50 g/day increment* |  |  | *0.80 (0.48-1.33)* |
| Sugar (g/day) (median, range) |  |  |  |
| Q1: 98 (44-115) | 127 | 32 | 1.0 |
| Q2: 124 (115-132) | 126 | 11 | 0.35 (0.15-0.81) |
| Q3: 141 (132-151) | 128 | 24 | 1.07 (0.52-2.22) |
| Q4: 173 (151-258) | 126 | 22 | 0.80 (0.36-1.77) |
| P-trend^b^ |  |  | 0.93 |
| *Per 50 g/day increment* |  |  | *0.98 (0.64-1.51)* |
| Fibre (g/day) (median, range) |  |  |  |
| Q1: 24 (11-27) | 127 | 28 | 1.0 |
| Q2: 30 (27-32) | 127 | 24 | 0.81 (0.40-1.64) |
| Q3: 35 (32-38) | 127 | 24 | 0.70 (0.33-1.46) |
| Q4: 43 (39-74) | 126 | 13 | 0.29 (0.12-0.67) |
| P-trend ^b^ |  |  | 0.005 |
| *Per 10 g/day increment* |  |  | *0.69 (0.50-0.97)* |

^a^ Multivariable Model: adjusted for age, education, BMI, smoking (pack years), physical

activity, lifetime mean alcohol intake, non-steroidal anti-inflammatory drug (NSAID) use, total

fruit intake (except for fiber intake), red meat, processed meat, and total energy

^b^ Likelihood ratio test for trend across dietary variables quartiles by using an ordinal variable

coded as the median value of the quartile
